# Cerebral Cavernous Malformation Associated With Left Middle Cerebral Artery (MCA) Aneurysm and Bilateral Internal Carotid Artery (ICA) Dissections

**DOI:** 10.7759/cureus.34715

**Published:** 2023-02-07

**Authors:** Akhil Padarti, Alexandria Penwell, Muhammad-Adeel Saleemi, Rebecca Sugg, Taha Nisar

**Affiliations:** 1 Department of Neurology, Frederick P. Whiddon College of Medicine, University of South Alabama, Mobile, USA

**Keywords:** magnetic resonance imaging, focal seizure without impairment of awareness, bilateral ica dissections, middle cerebral artery aneurysm, cerebral cavernous malformations

## Abstract

Cerebral cavernous malformations (CCMs) are the second most common type of cerebral vascular lesions. They are often associated with other vascular lesions, typically developmental venous anomalies. CCMs are not known to be associated with cerebral aneurysms and there is a paucity of literature on this occurrence. We report the case of a patient who presented with a focal seizure from a symptomatic CCM with acute hemorrhage and was incidentally found to have a cerebral aneurysm and bilateral internal carotid artery (ICA) dissections secondary to fibromuscular dysplasia. The presence of a cerebral aneurysm has clinical implications as these patients will need closer monitoring.

## Introduction

Cerebral cavernous malformations (CCMs), also known as cavernomas, are vascular malformations comprising dilated intracranial capillaries surrounded by a thin layer of endothelial cells. While these lesions can remain asymptomatic, they are also predisposed to intraparenchymal hemorrhages and seizures resulting in focal neurological deficits [1]. Cavernomas can exist as solitary lesions or mixed vascular lesions associated with other vascular anomalies. The most frequently associated venous anomaly is a developmental venous anomaly (DVA), seen in approximately 33% of all cavernomas. Unlike DVA, other associated lesions such as capillary telangiectasias and venous angiomas cannot be radiographically differentiated from CCM. Mixed vascular lesions have a higher propensity for hemorrhages than solitary lesions [2]. 

Cerebral aneurysms are dilatations within the arterial wall typically arising at a vascular bifurcation with variable morphology. The aneurysm wall is predisposed to rupture. Since these are high-flow vascular anomalies, rupture results in aneurysmal subarachnoid hemorrhage (SAH) associated with significant morbidity and mortality. These lesions need to be closely monitored for growth and possible surgical/endovascular treatment to prevent hemorrhage [3]. 

Here, we present a patient who had a symptomatic hemorrhage from a cortical CCM resulting in focal seizures. Incidentally, the patient was found to have an adjacent unruptured cerebral aneurysm and bilateral internal carotid artery (ICA) dissections. The hemorrhage was determined to be due to the CCM based on radiographic characteristics. The hemorrhage was deemed to be intraparenchymal rather than subarachnoid, which is more typical of CCM hemorrhage. There is limited literature on the association between CCMs and cerebral aneurysms [4]. This represents the second reported case of a CCM in conjunction with a cerebral aneurysm [5]. This association has clinical implications as cerebral aneurysm ruptures have devastating clinical consequences relative to CCM hemorrhages [1,3].

## Case presentation

**Figure 1:**
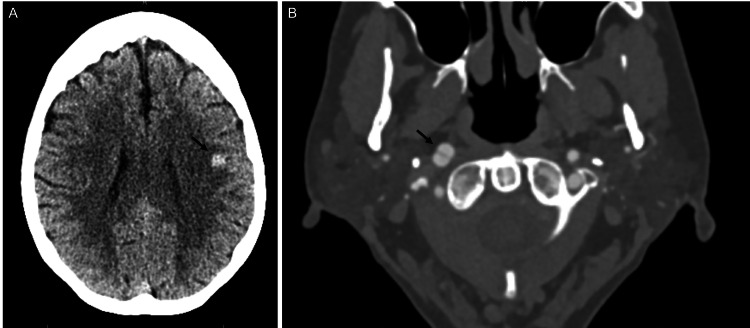
Initial CT imaging (A) Axial CT brain without contrast demonstrating left frontoparietal intraparenchymal hemorrhage; (B) Axial CT angiogram of the head and neck with contrast demonstrating dissecting pseudoaneurysm of the right internal carotid artery. Arrows are used where appropriate to indicate pathology.

**Figure 2:**
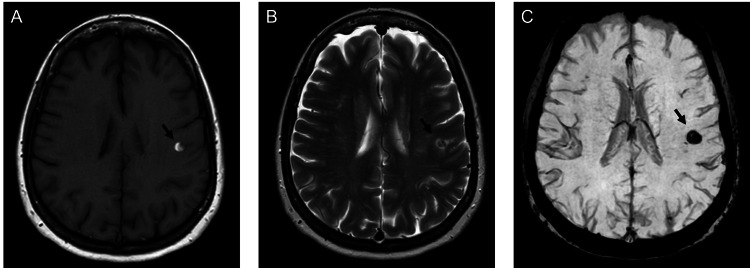
MRI of the brain obtained on 3 Tesla scanner (A) Axial T1 showing subacute hemorrhage in the left frontotemporal lobe; (B) Axial T2 showing heterogenous “popcorn” lesion with perifocal hemorrhage in the left frontotemporal lobe; (C) Axial susceptible weighted imaging showing blooming artifact in the left frontotemporal lobe. This is consistent with early subacute hemorrhage. Arrows are used where appropriate to indicate pathology.

Our patient is a 61-year-old Caucasian female who presented to the emergency department for acute onset of right facial pulling sensation followed by two intermittent episodes of right facial numbness lasting 30 minutes prior to presentation. She had a similar episode two years ago that lasted several days associated with right facial numbness. At that time, she was diagnosed with intraparenchymal hemorrhage (IPH), and she was lost to follow-up. Her physical exam was unremarkable. Her initial cranial imaging was significant for left frontoparietal IPH (Figure 1). CT angiography (CTA) head and neck showed dissections of the right ICA (Figure 1). MRI brain showed subacute left frontoparietal IPH with an underlying CCM (Figure 2). She was admitted to the ICU for close monitoring. No treatment was given. Her systolic blood pressure remained 140-150 mmHg without any medications. She was subsequently downgraded after 24 hours of clinical stability. She continued to have a normal neurological examination.

**Figure 3:**
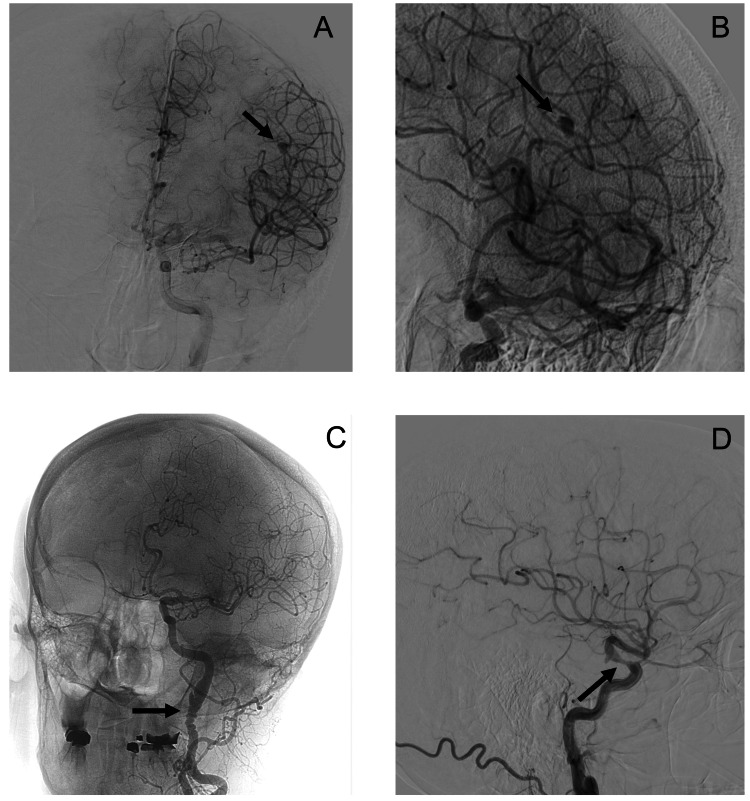
Diagnostic cerebral angiography (A) Anterior-posterior view of fusiform aneurysm of the parietal branch of the left middle cerebral artery; (B) Lateral view of fusiform aneurysm of the parietal branch of the left middle cerebral artery. Aneurysm measures approximately 5.4 mm x 2.8 mm; (C) Irregular appearance of the left internal carotid artery can be seen indicative of fibromuscular dysplasia. There is a dissection of the distal cervical segment of the left internal carotid artery with an intimal flap; (D) Dissection of the distal cervical segment of the right internal carotid artery with an intimal flap. Arrows are used where appropriate to indicate pathology.

Due to the atypical features of her angiographic imaging including the dissecting pseudoaneurysm in the right carotid artery, she underwent a diagnostic cerebral angiogram. The angiogram showed a 5.4 x 2.8 mm fusiform aneurysm of the left parietal branch of the left middle cerebral artery (MCA) (Figure 3). The patient was found to have a beading appearance of the bilateral cervical ICAs with associated bilateral non-occlusive internal carotid dissections. Interventional neurology deemed the patient was not a candidate for endovascular intervention due to the location and the angioarchitecture of the aneurysm. No intervention was deemed necessary for the asymptomatic bilateral carotid dissections. Neurosurgery was consulted, and no emergent intervention was indicated for the CCM. The risks of operative intervention of the CCM and cerebral aneurysm would outweigh the benefits given the minimal neurological symptoms. Her clinical presentation was determined to be a focal seizure without impairment of awareness, and she was started on levetiracetam, due to her high risk of recurrence.

Post discharge, the patient had a one-month follow-up appointment with interventional neurology and neurosurgery. A watchful waiting strategy was opted for by both services given the high operative risk. She will be followed by yearly clinical exams and repeat neuroimaging. She did not have any re-emergence of neurological symptoms.

## Discussion

Association of CCM and cerebral aneurysm

The simultaneous presence of CCM and cerebral aneurysm is a rare occurrence. This is the second published case in the literature. CCMs are the second most common cerebral vascular lesions with an incidence of one in 200-600, depending on the population surveyed [6-8]. CCMs are frequently seen with other venous anomalies such as DVAs and capillary telangiectasias. Cerebral aneurysms are frequently seen in feeder vessels of arteriovenous malformations (AVM). It is hypothesized that they form due to vascular flow changes that result from the AVM. Mixed vascular lesions are at increased risk of hemorrhage; however, it is unclear whether this would apply to lesions composed of CCM and cerebral aneurysm. Furthermore, our patient had fibromuscular dysplasia (FMD) of her ICAs resulting in a bead-like appearance. This is likely the cause of the patient’s bilateral nonocclusive ICA dissections [9,10]. There is a known association between FMD and cerebral aneurysms, but not CCMs [11]. Although it is still uncertain, our patient may have an underlying predisposition to vascular anomalies resulting in the development of a simultaneous CCM and cerebral aneurysm. It may be prudent to have a high clinical suspicion for cerebral aneurysms in patients with combined FMD and CCMs.

Determining the source of hemorrhage

In the presence of multiple arteriovenous anomalies, it may be difficult to ascertain the source of the hemorrhage. Both CCMs and cerebral aneurysms are associated with vessel rupture resulting in neurological deficits. In certain cases, the proximity of the hemorrhage to the vascular anomaly may be sufficient in determining the etiology. However, in our patient, both vascular anomalies were adjacent to the hemorrhage bed and, therefore, there is a potential for both lesions to cause the hemorrhage. This is not the case for other mixed vascular lesions composed of CCMs and DVAs, where DVAs are more susceptible to thrombosis than hemorrhage [12]. There are different clinical implications between aneurysmal rupture and CCM hemorrhage. Aneurysmal SAH has significantly higher morbidity and mortality than CCM hemorrhage [13]. In our case, the source of the hemorrhage was determined by radiographic imaging. The hemorrhage was more consistent with an intraparenchymal location as seen in CCMs than a subarachnoid location as seen in aneurysms. Therefore, it was agreed that the etiology of hemorrhage was due to the CCM. However, our patient is still at risk for aneurysm rupture and was counseled to recognize symptoms of aneurysmal SAH and to seek medical attention immediately.

Treatment considerations of combined CCM and cerebral aneurysm

There are certain treatment considerations in mixed vascular lesions composed of CCM and cerebral aneurysms. CCMs can be treated via surgical resection and radiotherapy. The decision for surgery is based on the symptomatic nature of the cavernoma and the location of the lesion [14]. This is typically a risk-and-benefit discussion between the neurosurgeon and the patient. In our case, the cavernoma was present in an eloquent area while the patient had minimal symptoms, and, therefore, a watchful waiting approach was elected. Cerebral aneurysms are treated via open vascular clipping and endovascular coiling [15]. Typically, endovascular is preferred due to the operative outcomes but may be unfeasible due to the location and the angioarchitecture of the aneurysm. In our case, the patient had a fusiform aneurysm of the left MCA branch. Both endovascular and surgical treatment can result in a potential sacrifice of the artery resulting in a stroke. Therefore, a conservative approach was opted for given the patient’s clinical stability. The patient will need to be followed for both vascular anomalies for possible intervention in the future; ideally, both lesions would be treated simultaneously to minimize the number of operations.

## Conclusions

CCMs are rarely associated with cerebral aneurysms. In cases with these mixed vascular lesions, it can be difficult to determine which is the etiology of the hemorrhage. Our patient was also found to have bilateral ICA dissections with an appearance that was consistent with FMD. Patients who present with CCMs may warrant additional evaluation for cerebral aneurysm, especially if they have simultaneous FMD. Optimal treatment for patients with these mixed vascular lesions remains to be an individualized decision between the patient and provider.
